# Reprogramming of cell fate: epigenetic memory and the erasure of memories past

**DOI:** 10.15252/embj.201490649

**Published:** 2015-03-27

**Authors:** Buhe Nashun, Peter WS Hill, Petra Hajkova

**Affiliations:** Medical Research Council Clinical Sciences Centre, Faculty of Medicine, Imperial College LondonLondon, UK

**Keywords:** cell fate, chromatin, induced pluripotent stem cells, reprogramming, transcription factors

## Abstract

Cell identity is a reflection of a cell type-specific gene expression profile, and consequently, cell type-specific transcription factor networks are considered to be at the heart of a given cellular phenotype. Although generally stable, cell identity can be reprogrammed *in vitro* by forced changes to the transcriptional network, the most dramatic example of which was shown by the induction of pluripotency in somatic cells by the ectopic expression of defined transcription factors alone. Although changes to cell fate can be achieved in this way, the efficiency of such conversion remains very low, in large part due to specific chromatin signatures constituting an epigenetic barrier to the transcription factor-mediated reprogramming processes. Here we discuss the two-way relationship between transcription factor binding and chromatin structure during cell fate reprogramming. We additionally explore the potential roles and mechanisms by which histone variants, chromatin remodelling enzymes, and histone and DNA modifications contribute to the stability of cell identity and/or provide a permissive environment for cell fate change during cellular reprogramming.

## Introduction

During the differentiation process, the developmental capacity of totipotent cells in the early embryo is progressively lost as these undertake cell fate decisions. This process is driven by the expression of cross-antagonistic transcription factors (TF) promoting development towards one cell fate while repressing an alternative differentiation path (Graf & Enver, 2009). Cell fate decisions are fortified by progressive acquisition of complex layers of epigenetic modifications at both the DNA and chromatin level (Goldberg *et al*, 2007; Xie *et al*, 2013; Ho *et al*, 2014). While cell identity is undeniably dictated by the expression profile guided by cell type-specific TFs (Davidson & Erwin, 2006), the robustness of the acquired transcriptional state is additionally crucially dependent on the configuration of the chromatin context in which these TFs operate (Voss & Hager, 2014). As the key epigenetic modifications acquired during developmental progression are stable and inherited through subsequent cell divisions, an ‘epigenetic memory’ is established that underlies the phenotypic stability of the differentiated cell state (Zhu *et al*, 2013; Jost, 2014; Shipony *et al*, 2014).

Although generally stable *in vivo*, cell fate decisions can be manipulated and even reversed, *in vitro*. The experimental demonstration that every cell of an organism contains the complete genetic information, and that the acquired somatic state can be reversed by exposing the somatic nucleus to the oocyte environment (Gurdon *et al*, 1958; Gurdon, 1960, 1962), set off a search for mechanisms implicated in the erasure of epigenetic memory and the re-establishment of pluri- or totipotency. It has subsequently been shown that cell identity is also amenable to reprogramming using cell fusion (Miller & Ruddle, 1976) and by overexpression of master regulator TFs (Davis *et al*, 1987). Ultimately, reprogramming of somatic cells back to pluripotency was achieved by the ectopic expression of (only) four TFs (Takahashi & Yamanaka, 2006).

In agreement with the role of TFs and gene regulatory networks in defining cell identity, reprogramming of cell fate requires extinction of the existing transcriptional programme followed by the establishment and stabilisation of the transcriptional network specific to the cell type of interest. It has, however, become increasingly obvious that the successful reprogramming process entails, and in fact requires, complete erasure of the existing somatic epigenetic memory followed by the establishment of a new cell type-specific epigenetic signature. Thus, although changes to cell identity can be achieved by ectopic expression of key TFs alone, the efficiency of conversion remains painfully low, with existing chromatin modifications constituting a well-described barrier to the reprogramming process (Mikkelsen *et al*, 2008; Pasque *et al*, 2012; Chen *et al*, 2013b; Gaspar-Maia *et al*, 2013; Sridharan *et al*, 2013).

Here we summarise the current knowledge regarding the complex relationship between chromatin structure and reprogramming of cell fate. We additionally consider whether epigenetic changes are secondary to the newly established transcriptional networks, or whether establishing a permissive chromatin template is a necessary—or potentially even sufficient—step for cell reprogramming to occur.

## Transcription factors and chromatin structure: a two-way relationship

In a model whereby TF cross-antagonism is the central mechanism by which cell fate is determined, cell fate transitions, such as those observed during de-differentiation and trans-differentiation events, are possible through the ectopic expression of the required cell type instructive TFs (Graf & Enver, 2009). The most extreme and best studied example of this is the direct reprogramming of somatic cells to induced pluripotent stem cells (iPSCs) through the ectopic expression of the pluripotency-associated TFs: Oct4, Sox2, Klf4, and Myc (OSKM) (Takahashi & Yamanaka, 2006). The expression of these TFs destabilises the transcriptional network of differentiated somatic cells and induces the expression of the embryonic stem (ES) cell transcriptional network that eventually leads to the establishment of an ES-like phenotype (Adachi & Scholer, 2012; Niwa, 2014).

In addition to changing the transcriptional network, overexpression of the OSKM transcription factors during iPSC reprogramming has been shown to induce large-scale chromatin changes that ultimately lead to the establishment of a chromatin template highly similar to that of ES cells (Orkin & Hochedlinger, 2011; Liang *et al*, 2012; Apostolou & Hochedlinger, 2013). Of note, the establishment of this chromatin template appears to be finely regulated by OSKM expression levels: sustained high transgene levels appear to hinder the proper establishment of specific (bivalent) chromatin marks during the later stages of iPSC induction, while establishment of the normal ESC-like epigenetic signature can be achieved upon lowering/attenuating expression of the four transgenes at an intermediate point during the induction process (Hussein *et al*, 2014; Tonge *et al*, 2014).

**Figure 1:**
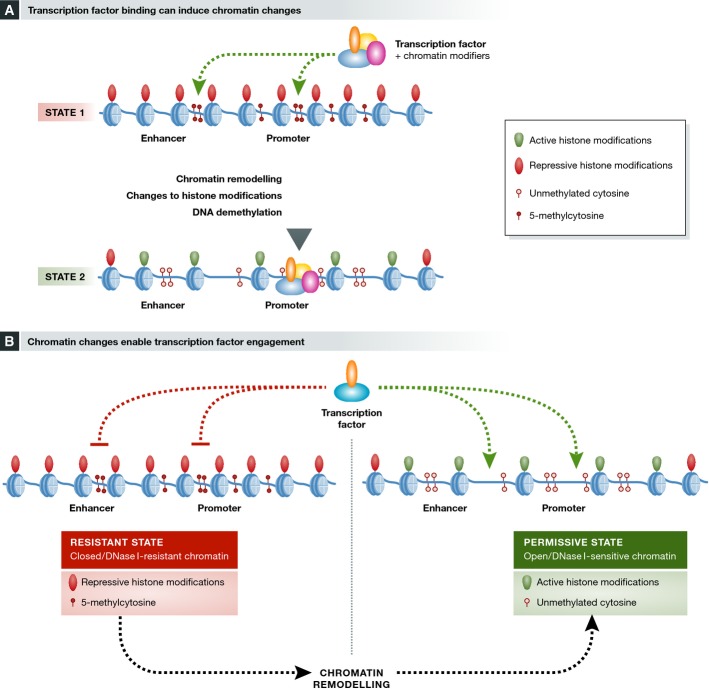
Relationship between transcription factors and chromatin configuration during cell reprogramming (A) Pioneer transcription factors (TFs) are known to reshape the chromatin landscape in the regions where they bind, both by enabling the binding of other TFs and through direct recruitment of various histone modifiers. In addition, the binding of both pioneer and non-pioneer TFs is known to induce locus-specific DNA demethylation. (B) Closed inaccessible chromatin in the original somatic cell type, marked by repressive histone modifications and DNA methylation, hinders the initial engagement of reprogramming-associated TFs. In turn, the activity of chromatin-modifying enzymes results in a permissive chromatin configuration that allows for fast and effective engagement of the introduced TFs, enabling efficient reprogramming.

In general, TFs (including OSK) are known to reshape the chromatin landscape in the regions where they bind, both by enabling the binding of other TFs and through direct recruitment of various histone modifiers (Mal & Harter, 2003; Ancelin *et al*, 2006; Magnani *et al*, 2011; Zaret & Carroll, 2011; Soufi *et al*, 2012; Drouin, 2014; Sherwood *et al*, 2014) (Fig1A). Moreover, the binding of TFs is known to induce locus-specific DNA demethylation (Stadler *et al*, 2011; Feldmann *et al*, 2013). In accordance with these observations, large-scale chromatin changes associated with iPS reprogramming may be a secondary phenomenon that follows destabilisation of the somatic transcriptional network and establishment of the new pluripotency network. The observed chromatin changes would thus not themselves be directly implicated in the reprogramming process, but rather would reflect successful establishment of the pluripotent state they are associated with.

Contrary to this view, accumulating evidence points towards an important role for chromatin in early stages of reprogramming. It has been shown that the initial engagement of OSK factors during iPS reprogramming is hindered by repressive histone modifications (Soufi *et al*, 2012), and the failure to successfully establish new gene regulatory networks in trans-differentiation experiments clearly correlates with the presence of closed inaccessible chromatin in the original somatic cell type (Cahan *et al*, 2014; Morris *et al*, 2014). Additionally, both repressive H3K9me2/3 histone methylation and the presence of 5mC have been documented to act as a barrier to the reprogramming process (Mikkelsen *et al*, 2008; Lister *et al*, 2011; Chen *et al*, 2013b; Sridharan *et al*, 2013). Considering these observations, efficient reprogramming appears to require an optimal chromatin configuration that not only allows for fast and effective engagement of the introduced TFs, but additionally promotes the exchange of chromosomal components, thus enabling fast and efficient erasure of pre-existing DNA and histone modifications (Fig1B).

## Reprogramming requires opening of the compacted somatic chromatin template

**Figure 2:**
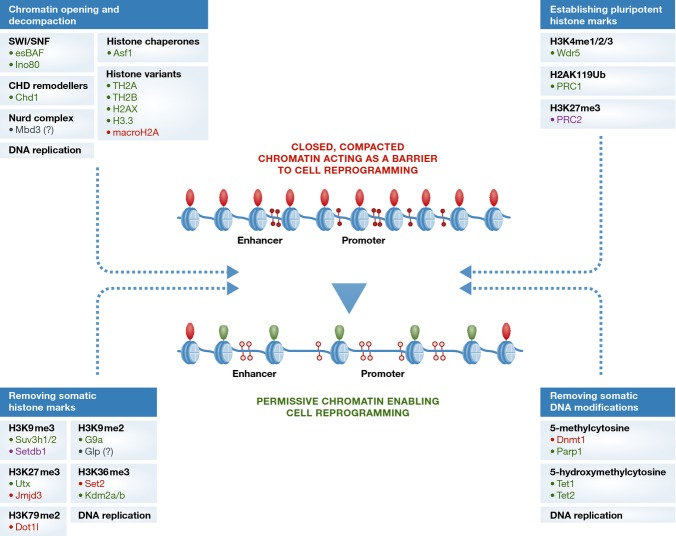
Chromatin components and modifiers affecting reprogramming efficiency Reprogramming requires the establishment of permissive chromatin and is associated with chromatin opening and changes to histone and DNA modifications. Multiple factors have been implicated in these processes: marked in green and red are factors whose presence/activity is associated with increased and decreased reprogramming efficiency, respectively; marked in purple are those factors whose presence/activity has been shown to both increase and decrease reprogramming efficiency in a context-dependent manner; factors whose influence on reprogramming requires further investigation are marked by (?).

Developmental progression from a totipotent to a differentiated cell is a gradual process accompanied by deposition of repressive histone marks and by increasing chromatin compaction (Gifford *et al*, 2013; Xie *et al*, 2013; Zhu *et al*, 2013). Successful iPS reprogramming thus requires removal of the somatic repressive chromatin to allow for conversion to a highly dynamic pluripotent chromatin state that is largely devoid of heterochromatin (Meshorer *et al*, 2006). In agreement with this, accumulating evidence suggests that chromatin remodelling complexes and selective deposition or eviction of certain histone variants play important roles in the acquisition and subsequent maintenance of the permissive pluripotent chromatin state (Fig2 and Table1).

**Table 1:** The roles of chromatin modifiers during somatic cell reprogramming

Category	Chromatin modifiers	Roles in reprogramming	References
Histone modifications	H3K4me2/3	Marks promoters and enhancers of pluripotency- or differentiation-associated genes during initial steps of reprogramming	Ang *et al* (2011); Koche *et al* (2011)
	H3K9me2/3	Marks broad heterochromatin regions refractory to initial OSKM binding; acts as an epigenetic barrier towards reprogramming	Soufi *et al* (2012); Chen *et al* (2013b); Sridharan *et al* (2013); Matoba *et al* (2014)
	H3K27me3	Represses pluripotency-associated genes in somatic cells and differentiation-associated genes in iPSCs	Mansour *et al* (2012)
	H3K36me2/3	Marks promoter regions of early responsive (MET) genes and represses their activation	Liang *et al* (2012)
	H3K79me2	Marks transcriptionally active genes; acts as a barrier for efficient repression of lineage-specific genes	Onder *et al* (2012)
Heterochromatin proteins	HP-1γ	Impedes reprogramming by repressing Nanog reactivation	Sridharan *et al* (2013)
Histone modifiers	Wdr5	Enhances reprogramming by physically interacting with Oct4 and maintaining H3K4me3 on pluripotency-associated gene promoters	Ang *et al* (2011)
	SUV39H1/2	Enhances reprogramming by facilitating Oct4/Sox2 binding through H3K9me3 demethylation	Onder *et al* (2012)
	G9a	Inhibition or down-regulation of G9a enhances reprogramming by regulating global H3K9me2/3 levels	Ma *et al* (2008); Shi *et al* (2008); Chen *et al* (2013b); Sridharan *et al* (2013)
	Setdb1	(?) Down-regulation enhances reprogramming by facilitating H3K9me3 status at core pluripotency genes in one study while opposite effect was observed in another study	Onder *et al* (2012); Chen *et al* (2013b)
	Ehmt1	(?) Down-regulation enhances reprogramming by regulating global H3K9me2/3 levels in one study but opposite effect was observed in another study	Onder *et al* (2012); Sridharan *et al* (2013)
	PRC1 (Ring1, Bmi1)	Down-regulation of Ring1 or Bmi1 reduces reprogramming efficiency, while overexpression of Bmi1 enhances reprogramming efficiency by regulating H3K27me3 levels	Pereira *et al* (2010); Moon *et al* (2011); Onder *et al* (2012)
	PRC2 (Ezh2, Suz12, Eed)	Down-regulation of Ezh2, Suz12, or Eed reduces reprogramming efficiency, while overexpression of Ezh2 enhances reprogramming efficiency by maintaining H3K27me3 at lineage-specific genes	Pereira *et al* (2010); Buganim *et al* (2012); Onder *et al* (2012); Fragola *et al* (2013)
	Utx	Physically interacts with OSK; facilitates iPS formation by H3K27me3 de-methylation at pluripotency-associated genes	Mansour *et al* (2012)
	Jmjd3 (Kdm6b)	Depletion increases iPS generation efficiency while overexpression inhibits reprogramming through up-regulating *Ink4a/Arf* locus expression by H3K27me3 demethylation; also promotes degradation of PHF20 independent of its demethylase activity	Zhao *et al* (2013)
	Jhdm1a/b (Kdm2a/b)	(?) Down-regulation reduces reprogramming efficiency, while overexpression enhances reprogramming by activating early responsive (MET) genes and the expression of microRNA cluster *302/367*	Wang *et al* (2011); Liang *et al* (2012)
	Dot1L	Down-regulation enhances reprogramming by promoting the silencing of lineage-specific genes through loss of H3K79me2	Onder *et al* (2012)
Chromatin remodellers	MBD3/NuRD	Down-regulation enhances reprogramming by facilitating the reactivation of downstream OSKM target genes in one study, while opposite effect was observed in another study	Rais *et al* (2013); dos Santos *et al* (2014)
	Ino80	Down-regulation leads to more closed chromatin structure near pluripotency gene promoters and reduces reprogramming efficiency	Wang *et al* (2014b)
	Chd1	Down-regulation leads to accumulation of heterochromatin and reduces reprogramming efficiency	Gaspar-Maia *et al* (2009)
	BAF (Brg1, Baf155)	Brg1 and Baf155 synergistically increase reprogramming efficiency by enhancing Oct4 binding and facilitating de-methylation of *Oct4* and *Nanog* promoters	Singhal *et al* (2010)
Histone variants	H1foo	Overexpression maintains the pluripotency gene expression and maintains global low methylation status	Hayakawa *et al* (2012)
	H2A.X	Down-regulation of H2A.X completely inhibits iPS generation	Wu *et al* (2014)
	H3.3	H3.3 counteracts H1 binding, and down-regulation of H3.3 in oocyte leads to compromised somatic cell reprogramming	Braunschweig *et al* (2009); Wen *et al* (2014)
	macroH2A	Co-occupies pluripotency genes with H3K27me3 and acts as an epigenetic barrier to induced pluripotency. Down-regulation significantly enhances iPS generation	Pasque *et al* (2012); Barrero *et al* (2013); Gaspar-Maia *et al* (2013)
	TH2A/B	Co-overexpression enhances reprogramming by inducing an open chromatin structure	Shinagawa *et al* (2014)
Histone chaperones	ASF1A	Overexpression enhances reprogramming by increasing global H3K56ac levels in the presence of GDF9 in culture medium	Gonzalez-Munoz *et al* (2014)
DNA modifiers	Dnmt1	Inhibiting activity by small molecules or knockdown significantly increases reprogramming efficiency	Mikkelsen *et al* (2008)
	TET1/2	Physically interacts and acts in synergy with Nanog. Oxidises 5mC in *Oct4* regulatory elements, although the importance of this is unclear; induces TDG-mediated demethylation at the *mir200* cluster, which is necessary for MET during fibroblast reprogramming	Doege *et al* (2012); Costa *et al*, (2013); Gao *et al* (2013); Hu *et al* (2014)
	PARP1	Functions in the regulation of 5mC; promotes Oct4 accessibility to *Nanog* and *Esrrb* loci	Doege *et al* (2012)
	Dnmt3a/b	Dispensable for nuclear reprogramming of somatic cells to pluripotent state	Pawlak and Jaenisch (2011)

### Chromatin remodelling factors

Multiple chromatin remodelling factors have been shown to regulate both ES cell identity and somatic cell reprogramming by their chromatin shaping activities. Of the SWI/SNF family of chromatin remodelling factors, esBAF (Brm/Brg-associated factor in ES cells) and Ino80 (inositol requiring 80) have been shown to be important both for the maintenance of ES self-renewal and pluripotency, and also for iPSC reprogramming (Ho *et al*, 2009b, 2011; Wang *et al*, 2014b). In ES cells, esBAF, as well as Ino80, co-localise genome-wide with the pluripotency factors (Ho *et al*, 2009a; Singhal *et al*, 2010; Wang *et al*, 2014b). The activity of these remodelling complexes leads to the generation of open chromatin structure and is thought to promote binding and transcriptional activity of the OSKM factors during reprogramming (Singhal *et al*, 2010; Wang *et al*, 2014b). In a similar manner, the CHD (chromodomain helicase DNA binding) family remodelling factor, Chd1, is also required to maintain open chromatin in ES cells and has been shown to be important for ES cell self-renewal and pluripotency (Gaspar-Maia *et al*, 2009). Down-regulation of Chd1 leads to accumulation of heterochromatin and significantly reduces reprogramming efficiency (Gaspar-Maia *et al*, 2009). These results thus collectively indicate that the potential to open chromatin, or to maintain a less compacted chromatin state, is a prerequisite for the acquisition of pluripotency.

Contrary to the remodelling complexes implicated in the generation of open chromatin structure discussed above, the NuRD (nucleosome remodelling deacetylase) complex contains histone deacetylase activity implicated in gene repression. In the absence of Mbd3, one of the core subunits of the complex, embryonic stem cells exhibit LIF-independent self-renewal capacity associated with elevated expression of pluripotency-related genes (Kaji *et al*, 2006; Reynolds *et al*, 2012). Upon differentiation, Mbd3-null ES cells fail to fully repress genes that are expressed in pre-implantation embryos, which in turn leads to deficiency in lineage commitment (Kaji *et al*, 2006). Interestingly, Mbd3 depletion dramatically increases reprogramming efficiency and results in deterministic and synchronised iPSC reprogramming (Rais *et al*, 2013), even in the absence of c-Myc or Sox2 (Luo *et al*, 2013). It has been suggested that Mbd3/NuRD is recruited through direct interaction with OSKM transcription factors to downstream OSKM target genes and counteracts their reactivation during iPS induction. In the absence of Mbd3, this inhibitory effect is relieved, favouring re-activation of pluripotency genes and leading to improved reprogramming efficiency (Rais *et al*, 2013). However, another recent study reported that Mbd3/NuRD is required for efficient iPS generation from neural stem cells (NSC), pre-iPS cells and epiblast-derived stem cells (EpiSCs) (dos Santos *et al*, 2014). Although overexpression of Mbd3/NuRD does not have any positive or negative effect on iPSC induction efficiency, combined overexpression with Nanog improves both reprogramming kinetics and efficiency, which is in stark contrast with previous reports showing that overexpression of Mbd3 inhibits iPSC induction (Luo *et al*, 2013; Rais *et al*, 2013). The reported difference may be due to different induction methods and culture conditions used in these studies; however, further investigation is required to clarify the exact role of Mbd3/NuRD in iPSC generation.

### Histone chaperones and variants

In support of the idea of open chromatin structure promoting reprogramming, overexpression of the histone chaperone Asf1a favours the maintenance of ES cell pluripotency and enhances iPS induction efficiency from human adult dermal fibroblasts (hADFs). Asf1 (anti-silencing factor 1A) non-selectively binds to an H3-H4 heterodimer and facilitates its import from the cytoplasm into the nucleus thus directly regulating the availability of H3-H4 dimer for turnover by the canonical histone H3.1/2-chaperone Caf-1 or by the H3.3-chaperone Hira (Burgess & Zhang, 2013). Asf1a is also essential for acetylation of newly synthesised H3 at lysine 56 (H3K56ac) (Burgess & Zhang, 2013; Gonzalez-Munoz *et al*, 2014), and it has been suggested that Asf1a regulates the expression of core pluripotency genes during reprogramming by increasing global H3K56 acetylation levels (Gonzalez-Munoz *et al*, 2014).

The incorporation of various histone variants into nucleosomes has a marked impact on local chromatin structure and dynamics. In the context of iPSC reprogramming, combined over-expression of the histone variants TH2A and TH2B, which are normally enriched in the oocyte and early embryo (Montellier *et al*, 2013; Shinagawa *et al*, 2014), has been shown to enhance the efficiency of iPS generation ninefold. This effect is further enhanced by additional overexpression of the phosphorylation-mimic form of nucleoplasmin (P-Npm), a factor implicated in chromatin remodelling and zygotic gene activation following fertilisation (Shinagawa *et al*, 2014). Increased DNase I sensitivity upon forced expression of TH2A and TH2B and the synergistic effect of P-Npm suggests that the enhancement of somatic cell reprogramming occurs through the induction of an open chromatin structure (Shinagawa *et al*, 2014).

Similarly, histone variant H3.3 counteracts linker histone H1-mediated chromatin compaction, keeping diverse genomic sites in an open chromatin conformation (Braunschweig *et al*, 2009). H3.3 incorporation into donor nuclei is required for successful somatic cell nuclear transfer (SCNT) (Nashun *et al*, 2011; Jullien *et al*, 2012; Wen *et al*, 2014), and down-regulation of histone H3.3 in mouse oocytes leads to compromised reprogramming efficiency (Wen *et al*, 2014). This appears to parallel the *in vivo* situation, where, following fertilisation, the selective incorporation of H3.3 into the paternal genome by the H3.3-specific histone chaperone Hira is essential for its de-condensation (Inoue & Zhang, 2014; Lin *et al*, 2014), and loss of H3.3 leads to over-condensation during early embryonic development (Lin *et al*, 2013). While Hira-mediated H3.3 deposition is required for proper establishment of H3K27me3 at the promoters of developmentally regulated genes in embryonic stem cells, depletion of H3.3 or Hira has only minor transcriptional effects (Banaszynski *et al*, 2013). The role of these factors during induction of pluripotency remains largely unknown.

In comparison with TH2A, TH2B, and H3.3, macroH2A, with its unique macro-domain, is associated with a repressive chromatin state. In agreement with the open chromatin structure found in pluripotent cells, the pluripotent state is associated with low macroH2A levels that increase following cell differentiation (Creppe *et al*, 2012). MacroH2A is abundant in differentiated somatic cells, but disassociates immediately from somatic donor chromosomes during SCNT (Chang *et al*, 2010). Recent studies indicated that macroH2A acts as an epigenetic barrier to induced pluripotency: the absence of this particular histone variant enhances iPSC reprogramming up to 25-fold (Pasque *et al*, 2012), while its overexpression prevents efficient reprogramming of epiblast stem cells to naïve pluripotency (Pasque *et al*, 2012; Barrero *et al*, 2013). It has additionally been shown that macroH2A and H3K27me3 co-occupy the regulatory regions of pluripotency genes in somatic cells (Barrero *et al*, 2013; Gaspar-Maia *et al*, 2013). Although iPSCs induced in the absence of this histone variant are able to differentiate, they retain the ability to return to a stem cell-like state (Gaspar-Maia *et al*, 2013) likely due to the incomplete inactivation of pluripotent genes during differentiation (Creppe *et al*, 2012).

Recent reports have shed new light on a possible role of another H2A histone variant in the reprogramming process. Ectopic expression of reprogramming factors increases the level of phosphorylated histone H2A.X, and high basal levels of γ-H2A.X have been observed in both iPSCs and ESCs, decreasing upon differentiation (Banath *et al*, 2009; Turinetto *et al*, 2012). Depletion of H2A.X reduces the efficiency of iPSC derivation (Wu *et al*, 2014) and compromises self-renewal activity in ES cells (Turinetto *et al*, 2012). Although typically associated with the DNA damage response, high γ-H2A.X levels do not correlate with elevated levels of DNA damage response proteins (Turinetto *et al*, 2012). Thus, while these recent findings suggest that they play an important role during reprogramming, the exact mechanism by which H2A.X or its phosphorylated form (γ-H2A.X) contribute to self-renewal and iPSC reprogramming requires further investigation.

## Changes in histone post-translational modification linked to the reprogramming process

### Early iPS reprogramming is marked by rapid acquisition of active post-translational histone modifications

Rapid genome-wide changes of H3K4me2 distribution are one of the earliest events observed in the initial phase of reprogramming (Koche *et al*, 2011). H3K4me2 peaks exhibit dramatic changes at promoter and enhancer regions of more than a thousand genes, including both pluripotency-related and developmentally regulated loci. As positive H3K4me2 changes are observed on both pluripotent and developmentally regulated genes (including those expressed in MEFs), the observed initial histone modification changes thus likely predominantly reflect chromatin accessibility. Interestingly (and in line with above), H3K4me2 is targeted to the pluripotency-associated genes before their transcriptional activation. Wdr5, the key component of Set/MLL histone methyltransferase complex responsible for H3K4 methylation, has been shown to directly interact with Oct4 (Ang *et al*, 2011) and promoters gaining H3K4me2 are significantly enriched for targets of Oct4 and Sox2 (Koche *et al*, 2011). This interaction thus possibly explains the rapid acquisition of H3K4 methylation early during iPSC reprogramming at loci bound by ectopic Oct4. Consistently, Wdr5 is required not only for ES cell self-renewal but also for efficient reprogramming of somatic cells to pluripotency (Ang *et al*, 2011).

### Erasure and remodelling of repressive histone modifications

Although the initial observed epigenetic changes during the reprogramming process are connected with the acquisition of transcriptionally permissive histone marks (see above), the cumulative evidence suggests that it is the erasure and remodelling of repressive histone modifications that constitute the true barrier to the reprogramming process.

#### H3K9me2/3

In stark contrast to H3K4me3-containing regions, broad chromatin domains enriched for repressive H3K9me3 are refractory to initial OSKM binding (Soufi *et al*, 2012). Reduction of H3K9me3 levels through down-regulation of methyltransferases Suv39H1&2 enhances Oct4 and Sox2 binding at these regions and increases reprogramming efficiency (Onder *et al*, 2012; Soufi *et al*, 2012). Consequently, H3K9me3-marked broad heterochromatin regions are considered as an epigenetic barrier during somatic cell reprogramming (Soufi *et al*, 2012; Chen *et al*, 2013b; Sridharan *et al*, 2013). In support of this, a recent publication has also documented an inhibitory role for H3K9me3 during reprogramming by SCNT (Matoba *et al*, 2014). It should be however noted that the role of H3K9me3 in iPSC generation is context dependent, as the downregulation of Setdb1, another H3K9me3 methyltransferase, has been reported to both facilitate and impede reprogramming (Onder *et al*, 2012; Chen *et al*, 2013b). In this context, it has been argued that H3K9me3 is important for silencing of lineage-specific genes; consistently, Setdb1 has been shown to repress trophectoderm-specific genes in ES cells (Yeap *et al*, 2009; Yuan *et al*, 2009).

Next to H3K9me3, reduction of H3K9me2 through knockdown or inhibition of G9a methyltransferase also favours somatic cell reprogramming both in transcription factor- and in cell fusion-based reprogramming systems (Ma *et al*, 2008; Shi *et al*, 2008; Chen *et al*, 2013b; Sridharan *et al*, 2013). Contrary to G9a, the role of Ehmt1/Glp (a binding partner of G9a) during iPSC generation remains controversial (Onder *et al*, 2012; Sridharan *et al*, 2013).

#### H3K27me3 and PRC2

Large regions of metazoan chromatin containing developmentally regulated genes are silenced by H3K27me3 catalysed by polycomb repressive complex-2 (PRC2). In agreement with the necessity to remove somatic heterochromatin patterns, loss of H3K27me3 is observed during the earliest stages of reprogramming yielding a transient open/primed chromatin state (Koche *et al*, 2011; Hussein *et al*, 2014; Tonge *et al*, 2014). The removal of this repressive histone mark is likely mediated by Utx, a JmjC-domain-containing enzyme that specifically mediates H3K27me2/3 demethylation (Klose *et al*, 2006). The importance of Utx in iPS reprogramming is highlighted by greatly reduced reprogramming efficiency and aberrant global H3K27me3 and H3K4me3 epigenetic profiles in the iPS cells generated from Utx-depleted mouse embryonic fibroblasts (Mansour *et al*, 2012). Furthermore, complete absence of Utx completely abolishes the ability of somatic cells to be reprogrammed back to the ground state of pluripotency (Mansour *et al*, 2012). While Utx physically interacts with OSK reprogramming factors and removes the repressive mark from pluripotency-promoting genes such as *Fgf4*, *Sall4,* and *Sall1*, Utx overexpression does not increase the efficiency of iPSC formation, suggesting that it does not represent a rate-limiting factor in the process (Mansour *et al*, 2012). In contrast to Utx, Jmjd3 (Kdm6b), another histone H3K27me3 demethylase, negatively regulates somatic cell reprogramming, highlighting the locus specificity and partially non-overlapping functions of these enzymes. Depletion of Jmjd3 is thought to reduce cell senescence by inhibiting Ink4a/Arf expression through maintenance of H3K27me3 levels at its promoter, enhancing both the kinetics and efficiency of reprogramming (Zhao *et al*, 2013).

Although the somatic pattern of H3K27me3 needs to be erased during the iPSC reprogramming, global loss of H3K27me3 through down-regulation of Eed (resulting in loss of all PRC2 complexes) leads to a severe decline in the efficiency of iPSC reprogramming (Fragola *et al*, 2013); thus, silencing through H3K27me3 appears indispensable for the establishment of iPSCs (Fragola *et al*, 2013). Consistent with this idea, overexpression of the PRC2 catalytic subunit Ezh2 enhances reprogramming efficiency (Buganim *et al*, 2012); down-regulation of other PRC2 complex components (Suz12 and Eed) significantly hinders iPSC generation (Onder *et al*, 2012); and additional subunits of PRC2 in mouse ES cells (Jarid2, Mtf2 and esPRC2p48) act synergistically to enhance OSK (Oct4/Sox2/Klf4)-mediated mouse embryonic fibroblast reprogramming (Zhang *et al*, 2011). Moreover, components of the PRC1 complex, Ring1a and Bmi1, are also required for efficient reprogramming, with the combined overexpression of Bmi1 and Oct4 sufficient to induce iPSCs from mouse fibroblasts (Moon *et al*, 2011). Finally, and of note, PRC1 subunit Ring1b and PRC2 subunit Ezh2 are also required for ES cells to efficiently reprogramme somatic cells in cell fusion-based systems (Pereira *et al*, 2010).

### Epigenetic changes linked to the memory of active transcriptional state

One of the key steps during the reprogramming process is the extinction of the initial somatic transcriptional programme. Although the expression of somatic genes is typically down-regulated early during reprogramming (Brambrink *et al*, 2008; Stadtfeld *et al*, 2008; Polo *et al*, 2012), stalled reprogramming intermediates often show incomplete silencing of the somatic programme suggesting that the maintenance of the original gene expression profile constitutes one of the hurdles in the reprogramming process. From the chromatin point of view, actively transcribed genes are characterised by Set2-mediated H3K36me2/3 and Dot1l-mediated H3K79me2 histone modification marks present in gene bodies (Nguyen & Zhang, 2011; Venkatesh *et al*, 2012). Down-regulation of either of these modifications by knockdown of the relevant histone methyltransferase (Onder *et al*, 2012) or by an overexpression of the relevant histone demethylase (Wang *et al*, 2011; Liang *et al*, 2012) prior to the iPS reprogramming significantly enhances the reprogramming process. On the molecular level, removal of these histone marks leads to the efficient down-regulation of the original somatic transcription profile, thus promoting cell fate change.

## DNA modifications: 5-methylcytosine, 5-hydroxymethylcytosine and higher oxidative products during reprogramming

### Faithful reprogramming requires establishment of the pluripotent methylome

In contrast to the high levels of DNA methylation consistently observed in somatic cells, DNA methylation levels are low in the naïve pluripotent cells both *in vivo* (Mayer *et al*, 2000; Oswald *et al*, 2000; Smith *et al*, 2012, 2014; Guo *et al*, 2014; Wang *et al*, 2014c) and *in vitro* (Ficz *et al*, 2013; Habibi *et al*, 2013; Leitch *et al*, 2013; Takashima *et al*, 2014). Considering this, it has been suggested that global DNA demethylation is a conserved and required feature of reprogramming events (Hill *et al*, 2014).

The functional relationship between faithful transcriptome and methylome reprogramming in iPSC and SCNT experimental systems has been recently shown by Mitalipov and colleagues (Ma *et al*, 2014). Using genetically matched starting somatic cells, the authors of this study used whole-genome bisulphite sequencing and RNA sequencing to extensively compare the DNA methylomes and transcriptomes of iPSC lines, ES cell lines generated through SCNT, and ES cell lines generated through traditional *in vitro* fertilisation (IVF) (Ma *et al*, 2014). They observed that both the DNA methylome and transcriptome of SCNT-derived ES cell lines, where the somatic nucleus was exposed to the cytoplasm of the host oocyte, were highly similar to that of ES cell lines derived through IVF. In contrast, iPSC reprogramming, involving only the ectopic expression of reprogramming TFs, generated cell lines with both significant differences in gene expression and high numbers of aberrantly methylated regions (Ma *et al*, 2014). The study revealed a strong correlation between incomplete reactivation of gene expression during reprogramming and high promoter methylation in iPSCs (Ma *et al*, 2014), suggesting that incomplete demethylation during iPSC reprogramming may be responsible for the observed incomplete transcriptional reprogramming.

### DNA methyltransferase activity inhibits reprogramming efficiency

Studies investigating the direct relationship between DNA methylation and reprogramming efficiency have revealed that inhibition of global DNA methyltransferase activity through addition of 5-azadC to growth medium or targeted knockdown of the maintenance DNA methyltransferase Dnmt1 greatly increases reprogramming efficiency (Mikkelsen *et al*, 2008) (Fig2 and Table1). In contrast, the reprogramming potential of somatic cells depleted of the *de novo* methyltransferases Dnmt3a and Dnmt3b appeared largely unaffected (Pawlak & Jaenisch, 2011). These observations suggest that, while maintenance of the somatic methylome is a barrier that must be overcome, *de novo* deposition of methylation is not a requirement for successful iPSC reprogramming. In fact, the observation that the large majority of differentially methylated regions (DMRs) between iPSCs and IVF ES cells do not overlap DMRs between donor somatic cells and IVF ES cells suggest that *de novo* DNA methylation may potentially contribute to the aberrant transcriptional profiles observed in iPSCs (Ma *et al*, 2014). Combined, these observations suggest that faithful reprogramming of the methylome may be a rate-limiting step to successful cell reprogramming. Consistent with such a model, deposition of H3K4me2 in the earliest stages of iPSC reprogramming only occurs at promoters that are already hypomethylated in the somatic nucleus, while acquisition of H3K4me2 at hypermethylated somatic promoters appears to be an event restricted to late stages of iPSC reprogramming, presumably following DNA demethylation at these regions (Koche *et al*, 2011).

### Context-specific requirement for Tet enzymes and oxidation of 5-methylcytosine oxidation in reprogramming

The recently discovered Tet family of oxygenases (Tet1-3), which catalyse the oxidation of 5-methylcytosine (5-mC) to 5-hydroxymethylcytosine (5-hmC), 5-formylcytosine (5-fC), and 5-carboxycytosine (5-caC) through iterative rounds of oxidation (Tahiliani *et al*, 2009; He *et al*, 2011; Ito *et al*, 2011), have been implicated in reprogramming processes *in vivo* and *in vitro*, as we have recently reviewed (Hill *et al*, 2014).

In the context of iPSC generation, Tet proteins were originally identified as key mediators of reprogramming, as depletion of Tet1 and Tet2 resulted in significantly reduced efficiency of iPSC colony formation (Doege *et al*, 2012; Chen *et al*, 2013a; Costa *et al*, 2013; Gao *et al*, 2013; Hu *et al*, 2014). While it was originally suggested that Tet1-mediated 5-hmC formation was required for Oct4 reactivation through demethylation of *Oct4* regulatory elements (Gao *et al*, 2013), later studies were unable to reproduce these results (Hu *et al*, 2014). Further investigation revealed that Tet proteins are only necessary for somatic cells to undergo the mesenchymal-to-epithelial transition (MET) during iPSC reprogramming (Hu *et al*, 2014). Tet1-3 triple knockout somatic cells of epithelial morphology, and fibroblasts acutely depleted of all three Tet proteins only following MET, could both be efficiently reprogrammed to iPSCs (Hu *et al*, 2014). Further characterisation of the involvement of Tet proteins during MET revealed that Tet2 mediates the oxidation of 5mC at the MET-regulating *mir200* microRNA cluster, resulting in DNA demethylation and expression of the relevant microRNAs (Hu *et al*, 2014).

By comparison, the role of Tet proteins and 5-hmC in other *in vitro* reprogramming systems is less well characterised. A requirement for Tet2 has been described for reactivation of the somatic pluripotency-associated genes *Oct4*, *Nanog*, and *Cripto* during cell fusion experiments, although the mechanism by which this is achieved, and whether this is dependent on 5-hmC formation, is still unclear (Piccolo *et al*, 2013). Similarly, oocyte-derived Tet3 has been implicated in demethylation and reactivation of the somatic *Oct4* promoter in SCNT experiments (Gu *et al*, 2011). More experimental work remains to be done to understand the relative importance of Tet proteins and 5-mC oxidation for both cell fusion and SCNT reprogramming systems.

## DNA replication and cell division: a window of permissive chromatin?

Considering the stability of heterochromatin and its restrictive role in the reprogramming process, it is important to consider that cells undergo dynamic cell cycle-associated chromatin changes with the existing chromatin structure disrupted by passage of the replication fork during S phase (MacAlpine & Almouzni, 2013). In view of this, studies investigating the effect of cell cycle and cell division on reprogramming can provide additional functional insights into the role of chromatin structure in the reprogramming process. Using cells in distinct stages of the cell cycle, Boiani and colleagues and Fisher and colleagues definitively identified DNA synthesis in the somatic nucleus as an essential requirement for reprogramming in both SCNT (Wang *et al*, 2014a) and cell fusion (Tsubouchi *et al*, 2013) experimental systems. Consistently, early analysis of iPSC reprogramming mechanisms revealed that increased cell division rates achieved through down-regulation of the p53/p21 pathway or over-expression of Lin28 markedly accelerated reprogramming (Hanna *et al*, 2009), suggesting that increased frequency of cell cycling is associated with accelerated iPSC reprogramming.

It remains to be fully understood why DNA synthesis is a pre-requisite for reprogramming by cell fusion or SCNT (Tsubouchi *et al*, 2013; Wang *et al*, 2014a), or why accelerated cell division decreases the latency time of iPSC reprogramming (Hanna *et al*, 2009). One potential hypothesis considers the complex nature of chromatin replication during S phase. As mentioned above, an immediate consequence of DNA replication (and consequently cell division) is the disruption of the existing chromatin structure by passage of the replication fork (Alabert & Groth, 2012; MacAlpine & Almouzni, 2013). For faithful re-establishment of the parental epigenome, a link must exist between the DNA replication fork and the factors that propagate DNA modifications, histone modifications, the correct incorporation of histone variants and other non-histone chromatin proteins (Alabert & Groth, 2012; MacAlpine & Almouzni, 2013). Although the mechanisms for maintenance of DNA methylation patterns are relatively well understood, the abundance of histone modifications seems to fluctuate with progression through the cell cycle (Bonenfant *et al*, 2007). It is thus conceivable that chromatin changes associated with S phase can provide a window of opportunity for the ectopic TFs to bind their response elements. Additionally, over the course of a number of cell divisions, minor stochastic disruptions to the epigenetic inheritance could result in additional loss of epigenetic memory. In the context of differentiated cell states with robust transcriptional networks, minor disruptions would not likely result in overt phenotypic changes. However, upon exposure of the somatic nucleus to the pluripotent TFs (either through ectopic expression of the iPSC reprogramming factors, cell fusion, or SCNT), errors in the maintenance of epigenetic information and aberrant DNA accessibility may facilitate the recruitment of pioneer factors to regions normally recalcitrant to their binding (Soufi *et al*, 2012), or, more generally, of non-pioneer pluripotency-associated TFs to their DNA targets (Sherwood *et al*, 2014).

## Conclusions: is a permissive chromatin template sufficient for reprogramming in the absence of ectopic expression of instructive transcription factors?

**Figure 3:**
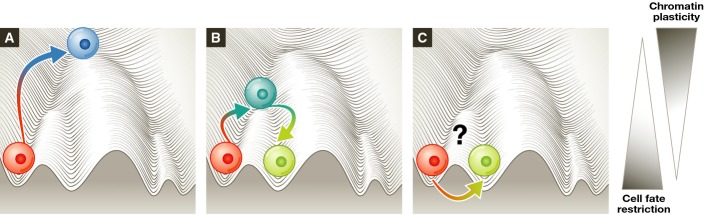
Is transition through a state characterised by open, dynamic chromatin a pre-requisite for all cell fate transitions? (A) Reprogramming to pluripotency appears to require increased chromatin plasticity. (B, C) Possible relationship between chromatin dynamics and trans-differentiation: (B) trans-differentiation via an upstream progenitor may be connected with a transient increase in chromatin permissiveness, and/or (C) direct trans-differentiation between two somatic states without transition through an intermediary state characterised by more plastic chromatin may be possible, although this has yet to be experimentally validated.

The model whereby the presence of cell type-specific TFs is the central mechanism by which cell fate is determined, and chromatin structure simply regulates the probability that TFs bind their genomic targets, suggests that reprogramming to pluripotency can only be induced when the somatic nucleus is exposed to the pluripotent TFs (through ectopic iPSC factor expression, cell fusion, or SCNT). According to this model, simple disruption of the underlying chromatin structure would be insufficient to drive reprogramming alone. Remarkably, however, it has recently been shown that full reprogramming of somatic cells can be achieved in the absence of forced TF overexpression through chemical manipulation of signalling pathways and epigenetic modifiers alone (Hou *et al*, 2013). It should be noted that reprogramming in the absence of instructive TFs is likely only possible when cells are being reprogrammed back to pluripotency, as trans-differentiation would ultimately depend on the presence of lineage specifying TFs. Additionally, the presence of specific culture conditions (e.g. agonists or antagonists of specific signalling pathways) potentially compensates in part for the absence of ectopic OSKM expression by providing a selective ‘environment’ during the reprogramming process. Nevertheless, with these caveats in mind, the ability to reprogramme somatic cells in the absence of instructive TFs clearly shows that it is possible to induce cell fate reversal by synergistically destabilising the chromatin template and the existing transcriptional network (using inhibitors of signalling pathways). Although most of our current understanding regarding chromatin dynamics during reprogramming stems from reprogramming back to pluripotency using the iPSC reprogramming system, the reached conclusions seem relevant also for trans-differentiation experiments, where manipulation of chromatin accessibility/dynamics might be an important factor to consider next to the establishment of the relevant gene regulatory network. In this context, trans-differentiation studies have documented that reprogramming somatic cells directly to a cell of another somatic fate involves de-differentiation and passage through a less differentiated progenitor state (Xie *et al*, 2004) (Fig3). While this progenitor state is likely to be characterised by a more plastic chromatin configuration, whether transitioning through a more open dynamic chromatin state during the intermediary steps of trans-differentiation is an absolute pre-requisite for cell fate change, or whether this appears to be a unique requirement for reprogramming to pluripotency, will have to be addressed in future work (Fig3).
